# Dysregulation in microRNA Expression Is Associated with Alterations in Immune Functions in Combat Veterans with Post-Traumatic Stress Disorder

**DOI:** 10.1371/journal.pone.0094075

**Published:** 2014-04-23

**Authors:** Juhua Zhou, Prakash Nagarkatti, Yin Zhong, Jay P. Ginsberg, Narendra P. Singh, Jiajia Zhang, Mitzi Nagarkatti

**Affiliations:** 1 Department of Pathology, Microbiology and Immunology, School of Medicine, University of South Carolina, Columbia, South Carolina, United States of America; 2 William Jennings Bryan Dorn Veterans Medical Center, Columbia, South Carolina, United States of America; 3 Department of Epidemiology and Biostatistics, Arnold School of Public Health, University of South Carolina, Columbia, South Carolina, United States of America; Duke University, United States of America

## Abstract

While the immunological dysfunction in combat Veterans with post-traumatic stress disorder (PTSD) has been well documented, the precise mechanisms remain unclear. The current study evaluated the role of microRNA (miR) in immunological dysfunction associated with PTSD. The presence of peripheral blood mononuclear cells (PBMC) and various lymphocyte subsets in blood collected from PTSD patients were analyzed. Our studies demonstrated that the numbers of both PBMC and various lymphocyte subsets increased significantly in PTSD patients. When T cells were further analyzed, the percentage of Th1 cells and Th17 cells increased, regulatory T cells(Tregs) decreased, while Th2 cells remained unaltered in PTSD patients. These data correlated with increased plasma levels of IFN-γ and IL-17 while IL-4 showed no significant change. The increase in PBMC counts, Th1 and Th17 cells seen in PTSD patients correlated with the clinical scores. High-throughput analysis of PBMCs for 1163 miRs showed that the expression of a significant number of miRs was altered in PTSD patients. Pathway analysis of dysregulated miRs seen in PTSD patients revealed relationship between selected miRNAs and genes that showed direct/indirect role in immunological signaling pathways consistent with the immunological changes seen in these patients. Of interest was the down-regulation of miR-125a in PTSD, which specifically targeted IFN-γ production. Together, the current study demonstrates for the first time that PTSD was associated with significant alterations in miRNAs, which may promote pro-inflammatory cytokine profile. Such epigenetic events may provide useful tools to identify potential biomarkers for diagnosis, and facilitate therapy of PTSD.

## Introduction

Post-traumatic stress disorder (PTSD) is a psychiatric disorder. Exposure to life-threatening events such as war, earthquake, sexual assault or family violence usually lead to PTSD [1]. It is estimated that 3.6% of American adults aged 18 to 54 years have PTSD. PTSD is often a chronic disorder in the combat Veteran population. For instance, about 30% of Vietnam Veterans developed PTSD [2]. Also, it was detected among 8% of Gulf War Veterans [3] and up to 35% of the military personnel deployed to Iraq and Afghanistan [4]. Patients with PTSD may display not only interpersonal withdrawal but also progressive decrease in muscle mass, increased risk of osteoporosis and fractures, as well as dementia due to tissue damage [5]–[7]. In addition, patients with PTSD are six times more at risk of committing suicide and having marital problems, and the annual loss of productivity is estimated to be approximately $3 billion [8]. Currently, there is no cure for patients with PTSD, and available treatments often are not completely effective.

Despite extensive research, the precise mechanisms of pathogenesis of PTSD are unclear. It has been reported that the biological dysfunction can result from immune alterations associated with PTSD [9]. There have been relatively few studies that have investigated immune functions in PTSD patients with contradictory findings. A recent review which analyzed published literature concluded that a majority of the reports indicated the presence of an excessive inflammatory state in PTSD, which may result from insufficient regulation by cortisol [10]. However, the role of immune system in the regulation or outcome of PTSD is still unclear [11].

Epigenetic regulation, involving the temporal and spatial control of gene activity without altering the basic structure of DNA, is getting increased attention in recent years. Epigenetic modifications can occur in response to environmental influences to alter the functional expression of genes [12]. Epigenetic regulation has been shown to play a major role in immune modulation and development and progression of diseases [13], [14]. Because PTSD patients are exposed to trauma, it is likely that epigenetic modifications may play an important role in the disease development and prognosis. Epigenetic regulation includes DNA methylation, histone deacetylation, chromatin remodeling and expression of microRNAs (miRNAs). miRNAs are small noncoding RNAs that regulate gene expression by binding to complementary target mRNAs and thus inhibit their translation. miRNAs have been implicated in immune regulation [15], [16]. There are no previous studies on epigenetic regulation of immune dysfunction by miRNA in PTSD. Elucidation of the role of miRNAs in PTSD may provide novel modalities for the effective care and treatment of PTSD patients and have great implications in understanding the pathogenesis of the disease.

In the current study, we determined the peripheral lymphocyte subsets, cytokine dysregulation and miRNA expression in patients with PTSD. Our studies demonstrate for the first time that PTSD patients exhibit pro-inflammatory T cell and cytokine profile, which correlates with specific changes in miRNA expression.

## Results

### Cell population changes in the blood of PTSD patients

**Figure 1:**
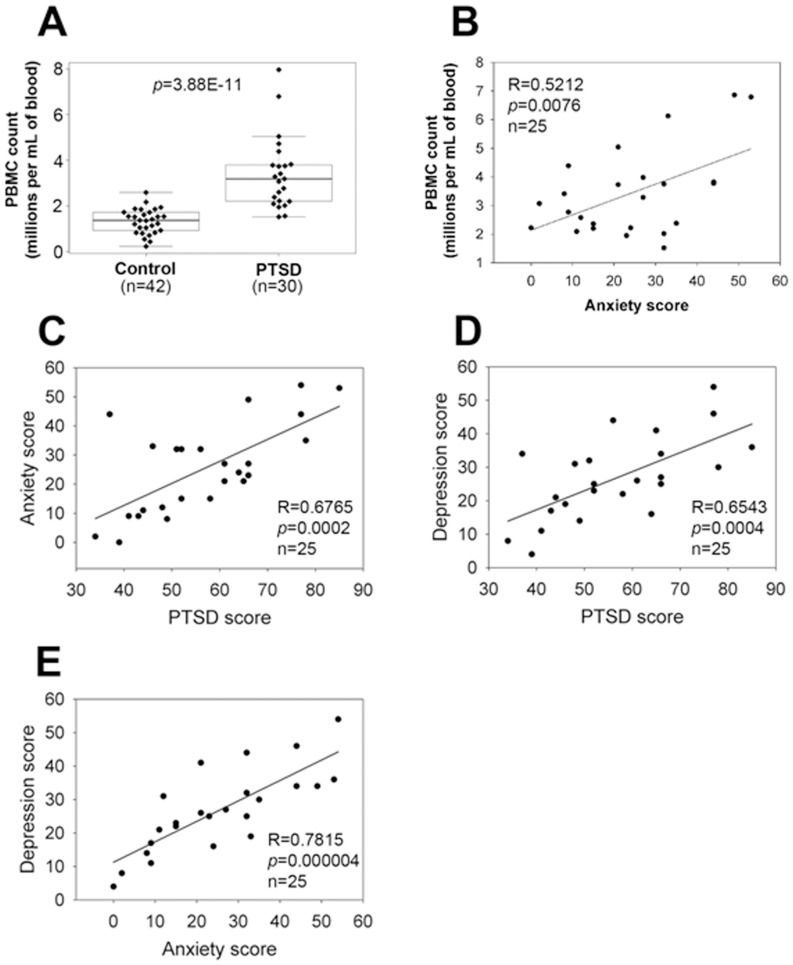
PBMC counts and correlation between clinical scores. The PTSD scores, anxiety scores and depression scores were used to determine the disease severity in PTSD patients. The higher score represented the more severe PTSD disease. PBMC were isolated from peripheral blood samples of patients with PTSD and healthy controls by Ficoll gradient centrifugation and viable cells were enumerated. Wilcoxon rank sum test was used to compare the difference of PBMC counts between controls and PTSD patients (**A**). Spearman rank correlation was used to analyze the correlation between anxiety scores and PBMC counts (**B**), between PTSD scores and anxiety scores (**C**), between PTSD scores and depression scores (**D**) and between anxiety scores and depression scores (**E**) in PTSD patients. Each dot or diamond symbol represents data from an individual patient or normal control. In panel **A**, the boxed data included 50% of measurements at medium level, the line in the box was the average value and the data beyond the upper line were the outliers. In panels **B–E**, both correlation coefficient (R) and *p* values were presented.

We first investigated the number of PBMCs found in patients with PTSD when compared to the controls. Table 1 lists the demographics and clinical history of the PTSD patients. Our analysis demonstrated that PBMC counts showed a significant increase in patients with PTSD when compared to the controls (Figure 1A). Furthermore, the PBMC counts in PTSD patients correlated with anxiety score [17], inasmuch as, increasing score was proportional to the PBMC counts (Figure 1B). Moreover, there was significant correlation among PTSD scores [18], anxiety scores and depression scores [19] (Figure 1C–1E).

**Table 1:** Demographics and clinical history of PTSD patients.

Patient code	Age (year)	Sex	Race	Anxiety score	Depression score	PTSD score	Medication
PCS2	40	Male	CA^1^	28	14	49	None
PCS3	30	Male	CA	28	30	64	4SSRI
PCS4	34	Male	AA^2^	19	36	57	Anti-histamine, 5NSA, Steroid
PCS8	34	Male	AA	34	34	74	Psychostimulant, 6SNRI
CS1	37	Male	CA	9	17	43	None
CS2	37	Male	CA	23	25	66	Opiate Analgesics, 7ACE inhibitor, Beta-blocker
CS3	44	Male	CA	32	45	56	NSA, Alpha-adrenergic blocker
CS4	34	Male	AA	53	36	85	None
CS5	45	Male	Hisp^3^	15	23	52	Anti-histamine
CS6	33	Male	AA	54	54	77	Tri-cyclic Anti-depressant, Opiate Analgesics, Anti-histamine, Benzodiazepine, NSA, Cholinergic
CS7	29	Female	Asian	19	26	61	Anti-histamine
CS8	31	Female	AA	8		49	Anti-histamine, Atypical anti-psychotic
CS9	33	Female	AA	27	27	66	SNRI
CS10	34	Male	AA	44	46	75	Muscle Relaxant, Anti-convulsant
CS11	54	Male	AA	32	32	51	None
CS12	46	Male	AA	9	11	41	Steroid
CS13	36	Male	AA	3	8	34	Muscle Relaxant
CS14	39	Male	AA	24	16	64	Opiate Analgesics, Anti-histamine
CS15	40	Male	AA				Opiate Analgesics, ACE Inhibitor, Anti-convulsant, Alpha-adrenergic blocker, Calcium Channel Blocker
CS16	42	Male	AA	35	50	78	Anti-histamine, Alpha-adrenergic blocker, Atypical anti-depressant
CS17	67	Male	AA	21	41	65	Beta-blocker, Alpha-adrenergic blocker
CS18	36	Male	CA	8	21	44	Anti-histamine, Atypical anti-depressant
CS19	43	Male	AA	12	31	48	None
CS20	32	Male	CA	44	34	37	None
CS21	41	Male	AA	49	34	66	Muscle Relaxant, Anti-histamine, Atypical anti-depressant
CS22	45	Male	CA	27		61	Tri-cyclic Anti-depressant, Opiate Analgesics, Anti-convulsant, NSA
CS23	43	Male	AA	33	19	46	Anti-histamine, Atypical anti-depressant
CS24	29	Male	CA	15	22	58	None
CS25	51	Male	CA	0	4	39	8SNDRI
CS26	49	Male	CA	17	26	39	None

Note:

1 CA, Caucasian American;

2 AA, African American;

3 Hisp, Hispanic;

4 SSRI, Selective serotonin re-uptake inhibitors;

5 NSA, Non-steroidal anti-inflammatory drugs;

6 SNRI, Serotonin-norepinephrine reuptake inhibitors;

7 ACE, Angiotensin-converting-enzyme;

8 SNDRI, Serotonin–norepinephrine–dopamine reuptake inhibitor.

**Figure 2:**
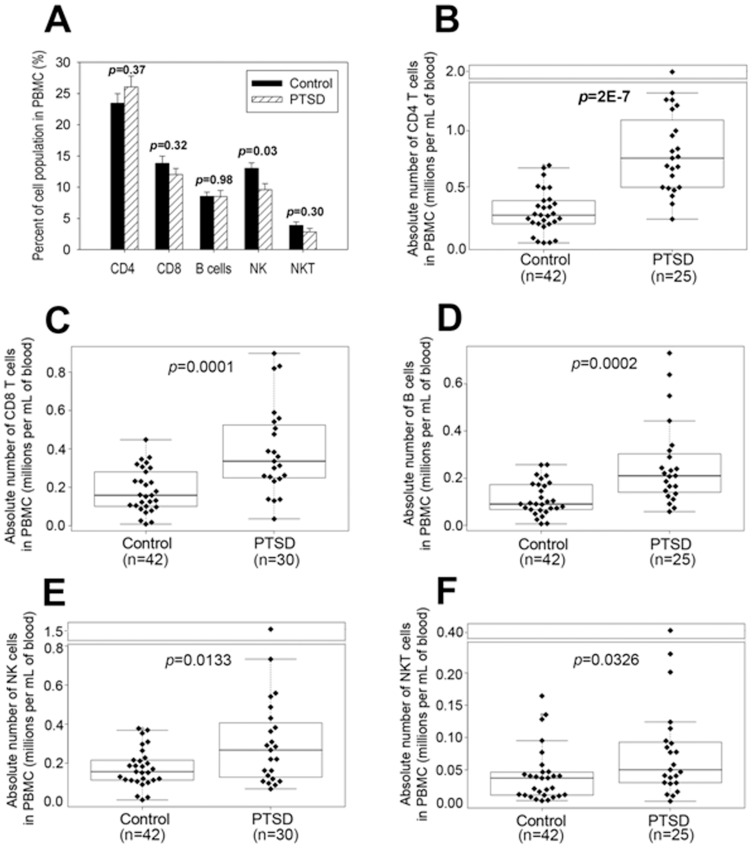
Comparison of cell populations in PBMC between controls and PTSD patients. PBMC were isolated from peripheral blood samples of patients with PTSD and normal controls as described in Fig. 1. After staining with FITC-anti-CD3, PE-anti-CD8 and APC-anti-CD4 antibodies, CD4 and CD8 T cells were analyzed by flow cytometry. After staining with FITC-anti-CD19 and APC-anti-CD3 antibodies, B cells (CD19+) were analyzed by flow cytometry. After staining with PE-anti-CD56 and APC-anti-CD3 antibodies, NK (CD56+) and NKT cells (CD56+CD3+) were analyzed by flow cytometry. The difference in the percentages of various lymphocyte subsets in PBMC between normal controls and PTSD patients was shown (**A**). The differences in absolute numbers of CD4 T cells (**B**), CD8 T cells (**C**), B cells (**D**), NK cells (**E**) and NKT cells (**F**) in PBMC between individual controls and PTSD patients were also presented. Wilcoxon rank sum test was used to compare the difference between controls and PTSD patients. In panels **B–F**, the boxed data included 50% of measurements at medium level, the line in the box was the average value and the data beyond the upper line were the outliers. *p* values were also presented.

Next, we investigated the percentage and absolute numbers of various lymphocyte subsets in the peripheral blood using flow cytometry. The profile of the percentages and absolute numbers of cells from all patients screened and controls has been shown in Figure 2. The data revealed that the percentage of various lymphocyte subsets did not change significantly in PTSD patients when compared to controls, except for NK cells where we noted a small but significant decrease in PTSD patients (Figure 2A). However, because the number of PBMCs increased in PTSD patients, when we calculated the absolute number of various lymphocyte subsets, we noted that the numbers of CD4+ T cells (Figure 2B), CD8+ T cells (Figure 2C), B cells (Figure 2D), NK cells (Figure 2E) and NKT cells (Figure 2F) increased significantly. Together, these results suggested that PTSD patients have marked increase in the numbers of most lymphocyte subsets.

### Alterations in T cell phenotypes in PTSD patients

**Figure 3:**
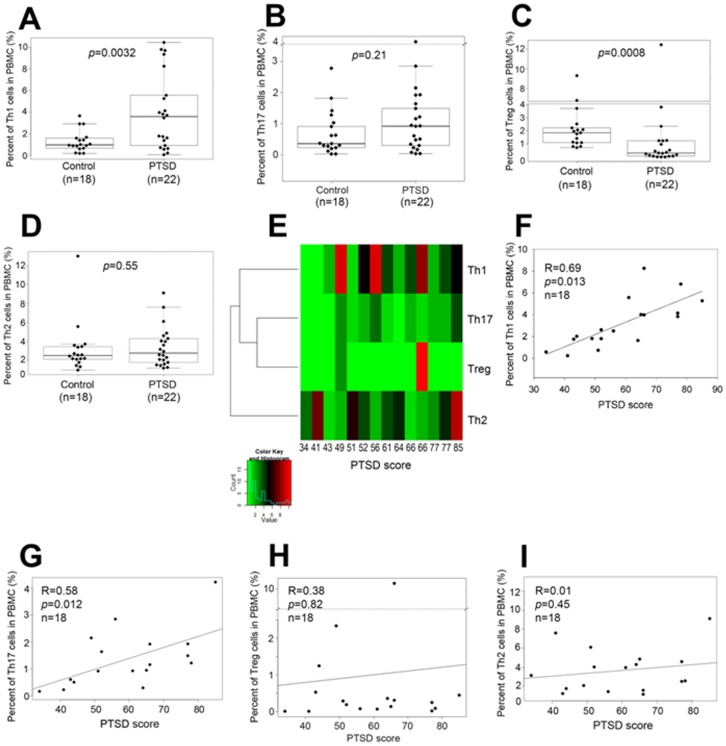
Comparison of T helper cell populations in PBMC between controls and PTSD patients and correlation between T helper cell populations and PTSD scores. **A–D:** Comparison of T helper cell populations in PBMC between controls and PTSD patients. PBMC from PTSD and normal controls were stimulated with 50 µg/mL of PHA for 3 days. Cells were stained with FITC-anti-IFN-γ, PE-anti-IL-4 and APC-anti-CD4 antibodies, and Th1 and Th2 cell populations were analyzed by flow cytometry. After staining with FITC-anti-IL-17A, PE-anti-FoxP3 and APC-anti-CD4 antibodies, Th17 and regulatory T cell (Treg) populations were analyzed by flow cytometry. Wilcoxon rank sum test was used to compare the difference of Th1 (**A**), Th17 (**B**), Treg (**C**) and Th2 (**D**) cell populations in controls and PTSD patients. The boxed data included 50% of measurements at medium level, the line in the box was the average value and the data beyond the upper line were the outliers. *p* values were also presented. **E–I:** Correlation between T helper cell populations and clinical scores in PTSD patients. PBMC from PTSD and normal controls were stimulated with 50 µg/mL of PHA for 3 days and stained for Th1, Th2, Th17 and Tregs as described above. Spearman rank correlation was used to analyze the correlation between PTSD scores and percentages of T helper cell populations from PTSD patients. The correlation between PTSD scores and T helper/Treg populations in PTSD patients was shown by the heat map (**E**), and the correlation between PTSD scores and Th1 (**F**), Th17 (**G**), Treg (**H**) and Th2 (**I**) cell populations in PTSD patients was presented. In panels **F–I**, correlation coefficient (R) and *p* values were presented.

Because we noted a significant increase in the absolute numbers of CD4+ T cells in patients with PTSD (Figure 2B), we next determined if they were T helper Th1, Th2, Th17 or Treg cells based on intracellular staining for IFN-γ, IL-4, IL-17 or FoxP3 respectively. We discovered that the percentage of IFN-γ-producing CD4+ T cells (Th1) (Figure 3A) increased significantly in PTSD patients and that of IL-17-producing CD4+ T cells (Th17) did not increase significantly (Figure 3B). In contrast, the percentage of FoxP3+ Tregs decreased (Figure 3C) while that of IL-4-producing CD4+ T cells (Th2) (Figure 3D) remained unchanged in PTSD patients. Furthermore, heat map analysis and Pearson's correlation test indicated that the increase in the percentage of Th1 cells (Figure 3E and 3F) but not Tregs (Figure 3E and 3H) and Th2 cells (Figure 3E and 3I) correlated with PTSD scores. Also, while the increase in the percentage of Th17 cells was not significant (Figure 3B), it correlated with PTSD scores (Figure 3E and 3G). Overall, these data suggested that PTSD patients have higher proportions of inflammatory Th1 cells and lower percentages of Tregs. However, only the increase in Th1 cells, which was statistically significant, correlated with PTSD scores.

### Cytokine dysregulation in PTSD patients

**Figure 4:**
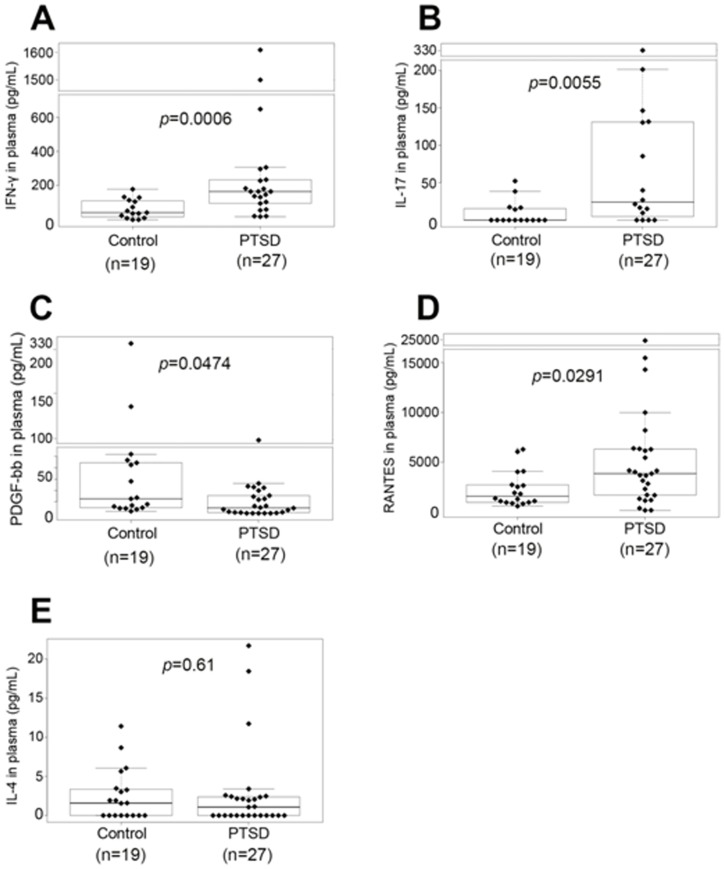
Comparison of cytokines IFN-γ, IL-17, PDGF-bb, RANTES and IL-4inplasma from controls and PTSD patients. Plasma samples were isolated from peripheral blood samples of patients with PTSD and normal controls by centrifugation. Then, Bio-Rad Bio-Plex method was used to determine the concentrations of multiple cytokines (including IL-1β, IL-1RA, IL-2, IL-4, IL-5, IL-6, IL-7, IL-8, IL-9, IL-10, IL-12(p70), IL-13, IL-15, IL-17, FGF-β, eotaxin, G-CSF, GM-CSF, IFN-γ, IP-10, MCP-1, MIP-1α, MIP-1β, PDGF-BB, RANTES, TNF-α and VEGF). Wilcoxon rank sum test was used to compare the difference of cytokines in the plasma between controls and PTSD patients. Plasma levels of IFN-γ (**A**), IL-17 (**B**), PDGF-bb (**C**), and RANTES (**D**) were found to be significantly different between controls and PTSD patients, whereas other cytokines such as IL-4 (**E**) did not have significant difference in plasma from controls and PTSD patients. The boxed data included 50% of measurements at medium level, the line in the box was the average value and the data beyond the upper line were the outliers. *p* values were presented.

We used Bio-Plex method [20] to determine the dysregulation of cytokines or chemokines (IL-1β, IL-1RA, IL-2, IL-4, IL-5, IL-6, IL-7, IL-8, IL-9, IL-10, IL-12(p70), IL-13, IL-15, IL-17, FGF-β, eotaxin, G-CSF, GM-CSF, IFN-γ, IP-10, MCP-1, MIP-1α, MIP-1β, PDGF-BB, RANTES, TNF-α and VEGF) in the plasma of PTSD patients. Of the cytokines screened, the levels of pro-inflammatory cytokines IFN-γ, IL-17 and RANTES increased significantly, but plasma levels of PDGF-bb significantly decreased in the PTSD patients when compared to normal controls (Figure 4A–D). We also noted that the levels of IL-4 did not change significantly in PTSD patients (Figure 4E) when compared to controls. These data together corroborated the Th cell analysis and indicated that PTSD patients had higher levels of pro-inflammatory cytokines such as IFN-γ and IL-17 in the plasma.

### miRNA expression in PTSD patients

**Figure 5:**
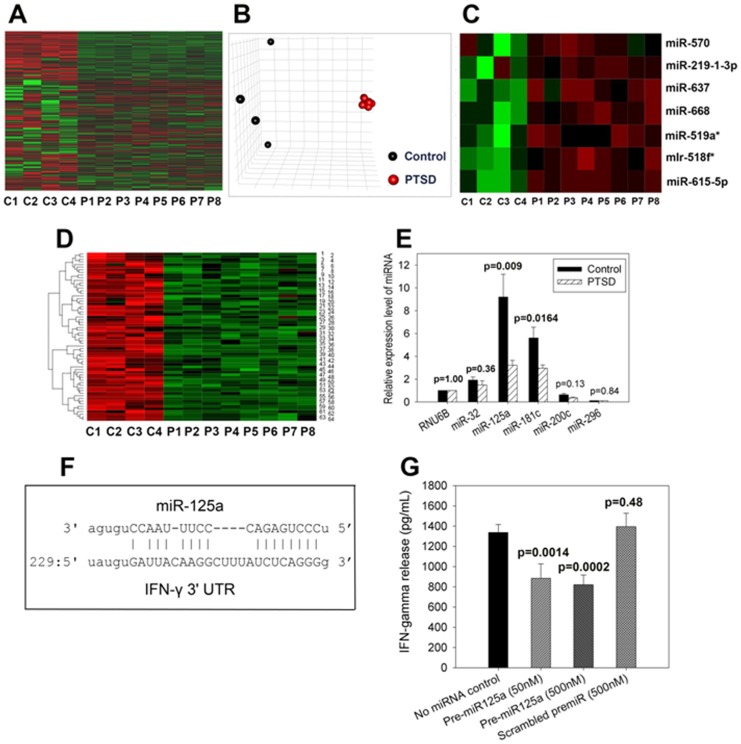
Dysregulation in miRNA expression profile in PBMC from PTSD patients and controls. **A–F:** Total RNA from PBMC of patients with PTSD and normal controls were used in the analysis of miRNA expression by Affymetrix miRNA array hybridization. Comparison of miRNA expression profiles between individual controls and PTSD patients was shown by the heat map (**A**) and plot of the principal component analysis accounting for 60.1% variability (**B**). Seven up-regulated miRNAs in PBMC from PTSD patients were compared with those from controls (**C**). 64 down-regulated miRNA molecules (>2.5 fold change, Table 1) in PBMC from PTSD patients were detected (**D**). Total RNA samples were also used in the confirmation of miRNA down-regulation (miR-125a and miR-181c) in PBMC from PTSD patients by real-time PCR (**E**). Wilcoxon rank sum test was used to compare the difference in miRNA expression in PBMC between controls and PTSD patients. **F–G:** Role of hsa-miR-125a in the regulation of IFN-γ. Complementary sequences between the seed sequence of miR-125a and 3′-untranslated region (3′UTR) of IFN-γ gene were compared (**F**). In silico studies were used to determine the complementary sequences between the seed sequence of miR-125a and 3′UTR of IFN-γ gene. The inhibitory effect of miR-125a on IFN-γ production in PBMC was determined (**G**). Hsa-miR-125a precursor (pre-miR125a) and pre-scramble control (scrambled premiR) were introduced into PBMC by electroporation. After PHA stimulation for two days, IFN-γ release from PBMC was determined in the culture supernatant by ELISA. Wilcoxon rank sum test was used to analyze the inhibitory effects of miR-125a on IFN-γ production in PBMC.

We next investigated if the increased production of pro-inflammatory T cells and cytokines were linked to differential expression of miRNAs. Using high-throughput miRNA microarray hybridization analysis, we investigated the expression of 1163 miRNAs in PBMC. The data shown in the Heat map (Figure 5A) and principal component analysis (PCA) plot (Figure 5B) demonstrated that miRNA expression in 8 PTSD patients, selected randomly, had a similar pattern as can be seen from the relatively dark green colors in all of the PTSD patients (Figure 5A) (suggesting lower expression levels) and were clustered in close proximity (Figure 5B), whereas those in 4 controls, selected randomly, were diversified (Figure 5A and 5B). Comparison of miRNA expression between control group and PTSD patient group revealed that 7 miRNAs were up-regulated in PTSD patients (Figure 5C) and as many as 64 miRNA were down-regulated 2.5 fold or more in PTSD patients (Figure 5D and Table 2). We used RT-PCR to validate the expression profile of 5miRNAs detected with miRNA array hybridization assay (Figure 5E) with or without significant change in expression between PTSD patients vs. controls, and corroborated that miRNAs such as miR-125a and miR-181c were significantly down-regulated in PTSD patients while others such as miR-32, miR-200c and miR-296 did not change significantly (Figure 5E).

**Table 2:** Down-regulated miRNAs (>2.5 fold changes) detected by miRNA array hybridization assay in patients with post-traumatic stress disorder.

No.	miRNA	Fold Change	Adjusted *p* value	No.	miRNA	Fold Change	Adjusted *p* value
1	miR-1281	−3.72	0.0162	33	miR-320d	−3.38	0.0005
2	miR-1280	−2.73	0.0306	34	miR-149*	−4.14	0.0007
3	miR-181c	−2.81	0.0176	35	miR-1228*	−3.04	0.0014
4	miR-1268	−3.62	0.0004	36	miR-26a	−2.89	0.0004
5	miR-584	−3.08	0.0417	37	miR-23a	−2.76	0.0165
6	miR-330-3p	−2.51	1.68E-05	38	miR-191	−2.91	0.0006
7	miR-23a*	−3.67	0.0062	39	miR-107	−2.63	3.24E-06
8	miR-27a*	−3.35	0.0017	40	let-7b	−3.40	0.0003
9	miR-199a-5p	−2.96	0.0389	41	miR-342-3p	−2.85	0.0119
10	miR-130a	−3.14	0.0080	42	miR-150	−2.97	0.0057
11	miR-1207-5p	−4.87	7.96E-06	43	miR-768-5p	−2.73	0.0109
12	miR-93*	−2.59	0.0001	44	miR-923	−7.62	0.0022
13	miR-197	−3.45	0.0045	45	miR-151-5p	−3.56	0.0116
14	miR-1308	−3.50	0.0015	46	miR-155	−3.01	0.0141
15	miR-200c	−2.58	1.11E-05	47	let-7g	−2.51	0.0004
16	miR-625	−2.71	0.0021	48	miR-361-5p	−3.05	0.0008
17	miR-145	−3.70	0.0030	49	miR-19b	−2.54	0.0002
18	miR-574-3p	−2.64	0.0043	50	miR-425	−2.67	0.0004
19	miR-199b-3p	−3.35	0.0483	51	miR-15b	−3.59	0.0034
20	miR-151-3p	−2.83	0.0442	52	let-7c	−3.91	0.0013
21	miR-30b	−2.52	0.0105	53	miR-221	−3.47	0.0005
22	miR-130b	−2.54	0.0002	54	miR-638	−3.23	7.71E-05
23	let-7f	−3.25	0.0018	55	miR-92a	−3.07	1.18E-06
24	miR-663	−2.77	0.0046	56	let-7d	−3.66	1.77E-08
25	miR-223	−3.58	0.0237	57	let-7a	−4.01	0.0017
26	miR-652	−2.58	0.0240	58	miR-1826	−4.01	0.0099
27	miR-25	−3.00	0.0002	59	miR-140-3p	−2.77	0.0086
28	miR-30c	−3.06	0.0002	60	miR-181a	−2.92	0.0084
29	miR-342-5p	−2.89	0.0230	61	miR-23b	−3.18	0.0319
30	miR-181b	−2.51	0.0204	62	miR-320c	−3.08	3.36E-05
31	let-7e	−3.15	0.0002	63	miR-320b	−2.91	0.0006
32	miR-125a-5p	−2.64	0.0127	64	miR-320a	−2.89	0.0004

### Effect of miR-125a on IFN-γ expression

It has been documented that miRNAs regulate cytokine gene expression [21]. Our in silico studies indicated that miR-125a, which was significantly down-regulated in PTSD patients, can bind to 3′-untranslated region (3′UTR) of IFN-γ mRNA (Figure 5F). Transfection experiments using PBMC from normal individuals confirmed that pre-miR-125a down-regulated IFN-γ production significantly (Figure 5G). These data suggested that in PTSD patients, down-regulated miR-125amay be responsible for increased production of IFN-γ by PBMC (Figure 4A), and increased proportion of Th1 cells in PBMC (Figure 3A).

### Analysis of miRNA expressed in PTSD patients

**Figure 6:**
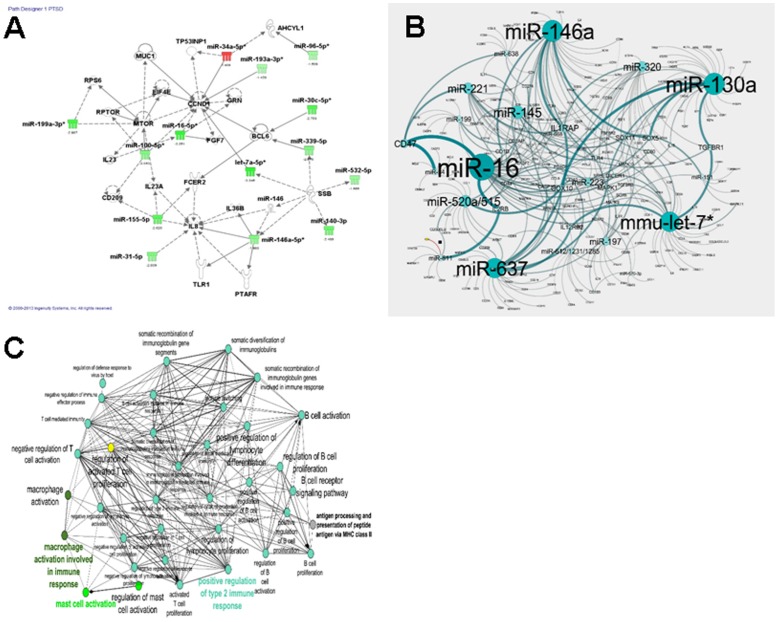
Pathway Analysis depicting a network of dysregulated miRNAs and target genes and their relationship in regulation of immune functions and pathways. **A.** Dysregulated miRNAs from PTSD patients were found to be involved in immunological pathways when Ingenuity Pathway Analyses were performed. The green tags represent down-regulated miRNAs and red tags up-regulated miRNAs in PBMC from PTSD patients when compared to controls. Circles represent genes, ovals are transcriptional regulators, diamonds are enzymes and triangles are kinases. Solid arrows represent direct action and dashed arrows indirect action. **B.** Depicts the relationship between miRNAs from PTSD patients with their target genes. First, both upregulated and downregulated miRNAs of PTSD patients were selected following Ingenuity Pathway analysis and then the relationship between miRNAs and genes were analyzed using Cytoscape software. Cytoscape analysis showed relationship between miRNAs and immunological pathways, especially between miRNAs and cytokine-associated genes (IL-1RA, IL-2, IL-2RB, IFN-γ, IL-12, IL-10, IL-17, IL-17R, TGF-β, IL-23A, etc.) playing a role in T cell development and immune functions. The larger circles represent miRNAs that are involved in regulation of a large number of genes, when compared to miRNAs represented by smaller circles. **C.** Shows the role of dysregulated miRNAs from PTSD patients in various immunological mechanisms and pathways. Upregulated and down-regulated miRNAs from PTSD patients were analyzed using Cytoscape (ClueGo) software. miRNAs from PTSD patients were found to be involved in several immunological and biological pathways.

We next used Ingenuity Systems Pathway Analysis to further analyze dysregulated miRNAs and their potential impact on the overall immune response in PTSD patients. There were several miRNAs and their target genes that showed direct or indirect relationship (Figure 6A). Of significance was the potential upregulation in IL-23 expression that plays a key role in Th17 induction which may explain increased production of IL-17 seen in PTSD patients. Also, several miRNAs showed a link to possible induction of IL-8, a proinflammatory chemokine.

In addition, we also selected 18 dysregulated (upregulated and downregulated) miRNAs for further broader analysis of immune response genes. The analysis using Cytoscape V.3.0.1 revealed relationship between selected miRNAs and genes that showed direct/indirect role in immunological signaling pathways that included a large number of CD molecules, cytokines, chemokines, early signaling molecules, apoptosis, MAP kinases, and the like (Figure 6B). Dysrgulated miRNA-associated genes were further analyzed using ClueGo analysis tool of Cytoscape which corroborated with the findings that the dysregulated miRNAs target a large number of immunological pathways including those involving T cells, B cells, macrophages, and mast cells (Figure 6C). Together, these data suggested that the dysregulation in miRNAs seen in PTSD patients may target a large number of pathways involving the complex regulation of the immune response, consistent with the immunological changes seen in these patients.

### Correlation between immunological changes and confounding factors

To investigate the role of confounding factors such as age, race, sex, anxiety, depression, and medications (Table 1), we performed statistical analysis including the Kruskal Wallis test as well as Pearson correlation test, and found that majority of alterations seen in PBMC numbers, cytokines, and miRNAs were independent of such confounding factors, while those that were dependent, such as on PTSD scores, were depicted as described above (Figures 1 and 3).

## Discussion

There have been relatively few studies that have investigated immune dysregulation in patients with PTSD. Increased numbers of inflammatory immune cells were reported in abused women with PTSD symptoms [22], as well as in male veterans with PTSD [23]. In contrast, other studies indicated either no significant change or immunosuppression in PTSD [24]–[26]. Overall, the general consensus has been that there is excessive inflammatory state in PTSD, which may result from insufficient regulation by cortisol, as indicated in a review [10]. In the current study, we noted that there was a significant increase in PBMC counts (Figure 1), the number of immune cells such as CD4+, CD8+, NK and B cells in PBMC (Figure 2), and the production of pro-inflammatory cytokines such as IFN-γ, IL-17 and RANTES (Figure 4) in PTSD patients. Furthermore, our studies provided the evidence for the first time that PBMC counts in patients with PTSD correlated with anxiety scores of PTSD patients (Figure 1B). These results suggested that PTSD patients are in an excessive inflammatory state.

It has been reported that Th1 cells are pro-inflammatory and play an important role in the pathogenesis of certain autoimmune diseases [27], [28], Crohn's disease [29] and certain cancers [30], [31]. The role of Th1 cell response in the pathophysiology of PTSD is unknown. Our study indicated that the percentage of Th1 cells increased significantly in PBMC from PTSD patients and this correlated with PTSD scores (Figures 3E and 3F), suggesting that PTSD was associated with Th1 response. In addition, we also noted that PTSD scores correlated with increases in Th17 cells. Interestingly, we did not see any significant impact of Th2 cells in these patients.

PTSD is a psychiatric disorder with abnormal and pathological fear and anxiety. Cytokines help convey to the brain of the presence of an infection in the periphery, and this action of cytokines can occur via the traditional endocrine route via the blood or by direct neural transmission via the afferent vagus nerve [32]. The fact that cytokines act in the brain to induce physiological adaptations that promote survival has led to the hypothesis that inappropriate, prolonged activation of the immune system may be involved in a number of pathological disturbances in the brain, including Alzheimer's disease, stroke and depression [32]. Thus, it is not surprising that cytokine abnormalities have been reported in PTSD patients [33], [34]. Individuals with primary DSM-IV PTSD had significantly elevated serum levels of IL-2, IL-4, IL-6, IL-8, IL-10 and TNF-α compared to age- and gender-matched healthy controls [35]. Plasma levels of IL-2, IL-4, and TNF-α were associated with PTSD [36]. Our analysis of 27 cytokines by Bio-Plex method [20], however, indicated that plasma levels of IFN-γ, IL-17 and RANTES significantly increased, but plasma levels of PDGF-bb decreased in PTSD patients (Figure 4). These results demonstrated that pro-inflammatory cytokines are dominant in the patients with PTSD, suggesting that these cytokines may play a role in the pathophysiology of PTSD.

Epigenetic mechanisms have recently been shown to play an important role in immune regulation, and consequently the development and progression of diseases [13]. Major epigenetic mechanisms include DNA methylation, covalent post-translational modifications of histone proteins, and small RNA-mediated gene silencing such as miRNA. A recent study suggested that global methylation pattern was increased along with altered methylation of CpG sites of genes associated with inflammation, in human subjects with PTSD [37]. However, there are virtually no earlier studies on epigenetic regulation of immune cells involving miRNA in PTSD. Our study analyzing 1163 miRNAs in PBMC by microarray hybridization assay demonstrated that some miRNAs were up-regulated, whereas many other miRNAs such as miR-125a and miR-181c were down-regulated in PTSD patients (Figure 5E and Table 2) when compared to healthy controls. Real-time PCR analysis confirmed that miR-125a and miR-181c were significantly down-regulated in PBMC from the patients with PTSD (Figure 5E). These results suggested the potential use of miRNA expression profile as signature biomarkers for PTSD. In silico analysis suggested that miR-125a targeted IFN-γ gene (Figure 5F), suggesting that miR-125a may inhibit IFN-γ production. Our study demonstrated that miR-125a down-regulated IFN-γ production (Figure 5G). Thus, it is possible that down-regulated miR-125a (Figure 5) in PBMC from PTSD patients may be responsible for IFN-γ up-regulation (Figure 4), and the increased level of IFN-γ may be responsible for induction of a Th1 cell increase in PBMC from PTSD patients (Figure 3). The Ingenuity Systems Pathway Analysis also showed how dysregulated miRNAs in PTSD patients may target and cause dysregulation in expression of IL-23 that plays a key role in Th17 induction, which may explain dysregulation in the expression of IL-17 in PTSD patients.

When we analyzed miR data using Cytoscape V.3.0.1 software, we found that the altered miRs may regulate several critical molecules involved in immunological signaling pathways such as CD molecules, cytokines, chemokines, early signaling molecules, apoptosis, MAP kinases, and the like (Figure 6B). These data combined with the analysis using ClueGo tool of Cytoscape confirmed that the dysregulated miRs in PTSD patients target a large array of cells and molecules of the immune system (Figure 6C). Together, these data suggested that miRNAs may play a crucial role in PTSD patients in modulating a large number of pathways involving the complex regulation of the immune response.

While the current study has performed extensive analysis of immunological changes and miRNA expression in PTSD patients, there are certain limitations: 1) While we ruled out any confounding factors that may have led to immunological alterations such as age, sex, race, depression score, and medication, it is possible that additional comorbidities may play a role. For example, symptoms of depression are often co-morbid with PTSD. For example, many of our subjects did have varying levels of depression as indicated by the scores (Table 1). While we noted no correlation between immunological changes and depression scores seen in these PTSD patients, additional studies are needed using patients with major depression without PTSD. 2) Combat Veterans with severe PTSD often have a history of alcohol and other substance abuse, immediately and for several months after return from deployment. For that reason, in the current study, we excluded recruitment of current alcohol and other substance abuse, defined as use within 6 months of recruitment. Thus, at the time of their participation in this study, none of the PTSD patients were actively abusing alcohol or drugs, which was also verified against medical record. Nonetheless, it is possible that alcohol and drug usage was under-reported by a Veteran. 3) It can be also be reasoned that excluding PTSD patients with substance or drug abuse leaves out a significant proportion of PTSD patients who may have shown similar immune profiles. However, it should be noted that only 3 Veterans were excluded initially for current/recent alcohol/drug abuse and 2 of them returned subsequently after the 6 month abstinence period was attained, and were enrolled, thereby leaving only 1 patient excluded in our study. It is not clear if potential immunological changes caused by alcohol/drug abuse persist over 6 months and whether they influence alterations associated with PTSD. Thus, additional studies are necessary on Veterans with alcohol and/or drug dependence with or without PTSD.

In summary, the current preliminary study demonstrates for the first time that combat Veterans clinically diagnosed with PTSD exhibit significant alterations in miR expression which correlates with immunological changes. Specifically we found that the PTSD patients had enhanced pro-inflammatory Th1 and Th17cytokine profile and decreased Tregs. These studies form the basis of future exploration to delineate if a single or unique signature miR profile in PTSD can be used towards biomarker identification and potential treatment.

## Materials and Methods

### Recruitment of normal control and PTSD patients

Healthy controls and PTSD patients were recruited for this study. All PTSD patients were combat Veterans returning from Persian Gulf, Iraq or Afghanistan war. Because many cases of severe PTSD also have a history of alcohol and other substance abuse, most often immediately and for several months after return from deployment, current alcohol and other substance abuse was excluded from the recruited sample. Thus, none of the PTSD patients in this study were actively abusing alcohol or drugs at the time of their participation. Alcohol and other substance abuse was defined as (a) substance abuse treatment within 6 months of enrollment, (b) any illicit drug usage, or (c) alcohol consumption greater than moderate (>2 drinks/day, whether beer, wine, or spirits). The screenings were checked against medical record. Subjects were assessed for severity of PTSD using the strategy recommended for diagnostic assessment of veterans for PTSD [38]. The diagnosis of PTSD was based on results of the Clinician Administered PTSD Scale (CAPS) [39] and the PTSD Checklist-Military Version (PCL-M) [18]. Interpretation of PCL-M scores was made using the following accepted test score categories: <17, no clinical PTSD; 17–33, low PTSD; 34–43, moderate PTSD; 44–85, high PTSD [40].

The lifetime co-morbidity of depression with PTSD is estimated to be 40–65% [41], [42]. To that end, we measured the severity of depression and non-traumatic anxiety according to the Beck Depression Inventory [43], [44] and Beck Anxiety Inventory [17], [45], respectively, which are two validated and widely used self-report clinical assessment instruments. For depression, the accepted test score categories were: 0–10, normal; 11–16, mild depression; 17–20, borderline clinical depression; 21–30, moderate depression; 31–40, severe depression; >40, extreme depression. The accepted test score categories for interpretation of the anxiety inventory were: 0–7, minimal anxiety; 8–15, mild anxiety; 16–25, moderate anxiety; 26–63, severe anxiety.

For normal controls, age-matched healthy volunteers, who did not have any psychiatric disorders or any symptoms of active infection or any history of immune compromise such as HIV, cancer, pregnancy or on chronic steroid therapy, were recruited. A total of 30 PTSD patients and 42 normal controls were recruited in this study, and the number of samples used in each assay is indicated in the Figure Legend. Ten mL of peripheral blood samples were drawn from PTSD patients and controls by hospital nurses using venipuncture and transferred into tubes coated with ethylenediaminetetraacetic acid (EDTA). Blood samples were processed immediately by Ficoll-Paque (GE Healthcare, Uppsala, Sweden) centrifugation to isolate PBMC samples. Viable PBMC were counted by trypan-blue exclusion.

### Ethics Statement

All healthy PTSD patients and healthy control subjects agreed to participate in this study by signing the William Jennings Bryan Dorn VA Medical Center Institutional Review Board (IRB) and Palmetto Health IRB-approved consent forms. Veterans visiting the William Jennings Bryan Dorn VA Medical Center with symptoms of PTSD were evaluated by the Psychometric properties of the PTSD Checklist (PCL) [18], and the PTSD diagnosis was validated by the Clinician Administered PTSD Scale [39]. IRB committee of University of South Carolina approved this study (IRB No: Pro00010512 and date of approval: 02-21-2013).

### Analysis of cell populations in PBMC

PBMCs were stained with FITC-conjugated anti-human CD3 or APC-conjugated anti-human CD3, APC-conjugated anti-human CD4, PE-conjugated anti-human CD8, FITC-conjugated anti-human CD19 and PE-conjugated anti-human CD56 monoclonal antibodies. Next, flow cytometric analysis was carried out using Cytomics FC500 flow cytometer (Beckman Coulter, Fullerton, CA) to determine CD4 T cells, CD8 T cells, B cells, NK cells and NKT cells in PBMC samples. All antibodies were purchased from Biolegend (San Diego, CA).

### PBMC stimulation in vitro and intracellular staining of helper T cells

PBMC were stimulated with 50 µg/mL phytohemagglutinin (PHA, Fisher Scientific, Pittsburgh, PA) in a humidified 5% CO_2_ incubator at 37°C for 3 days. Next, the culture supernatants were stored at −80°C for cytokine assay. PBMC were collected, fixed and permeabilized using Cytofix/Cytoperm (BD, Franklin Lakes, NJ) and stained with APC-conjugated anti-human CD4, FITC-conjugated anti-human IFN-γ or IL-17 and PE-conjugated anti-human IL-4 or FoxP3 monoclonal antibodies. IFN-γ, IL-4, IL-17 and FoxP3 producing CD4+ T cells were determined by flow cytometric analysis using Cytomics FC500 flow cytometer (Beckman Coulter, Fullerton, CA) to analyze Th1, Th2, Th17 and regulatory T (Treg) cells in PBMC samples, respectively. All antibodies were purchased from Biolegend (San Diego, CA).

### Bio-Plex Cytokine assay in plasma and culture supernatants

Twenty seven cytokines (including IL-1β, IL-1RA, IL-2, IL-4, IL-5, IL-6, IL-7, IL-8, IL-9, IL-10, IL-12(p70), IL-13, IL-15, IL-17, FGF-β, eotaxin, G-CSF, GM-CSF, IFN-γ, IP-10, MCP-1, MIP-1α, MIP-1β, PDGF-BB, RANTES, TNF-α and VEGF) in the plasma and culture supernatant samples were analyzed using Bio-Plex Pro Human Cytokine 27-plex Assay kit (Bio-Rad, Hercules, CA) according to the instruction manual. Briefly, the plasma or culture supernatant samples were mixed with the coupled beads in a 96-well filter plate and incubated on a shaker at room temperature for 30 min. After washing, the cytokine-coupled beads were mixed with the detection antibodies and incubated on a shaker at room temperature for 30 min. After washing again, the cytokine-coupled beads were mixed with streptavidin-PE and incubated on a shaker at room temperature for 10 min. After washing, the cytokine-coupled beads were resuspended in 125 µL of assay buffer. Next, data were acquired in the Bio-Plex Luminex 100 system and analyzed using Bio-Plex Manager Software version 5.0 (Bio-Rad, Hercules, CA) to determine 27 cytokines in plasma and cytokine production in supernatants of PBMC samples.

### miRNA array assays

Total RNA, including miRNA and other small RNA molecules, were isolated from randomized PBMC samples of PTSD patients and normal controls, using miRNeasy Mini kit and following the protocol of the company (Qiagen, Valencia, CA). The RNA integrity was verified using Agilent 2100 BioAnalyzer (Agilent Tech, Palo Alto, CA). Next, total RNA samples were subjected to miRNA array hybridization assay using Affymetrix miRNA-v1 gene chip according to the manufacturer's instructions. The miRNA array data were analyzed with Affymetrix miRNA QC tool software. The analysis pipeline detected the probe signals, estimated background and correction, normalized the signals and summarized the signal using median polish. The array performance was assessed using the quality control probes on the array and Pearson correlation of the control probes across the arrays. Fold changes in miRNA up-regulation or down-regulation were calculated using the formula: [Fold change = IF(X2> = 0, 2∧X2, −1/(2∧X2)), X = A-B, A = raw signal of specific miRNA from a PTSD patient, and B = raw signal of specific miRNA from a control], to compare the differences in1163 miRNAs expressed between PTSD patients and normal controls.

### Real-Time PCR of miRNA expression

Total RNA, including miRNA and other small RNA molecules, isolated from PBMC samples of PTSD patients and normal controls were used in cDNA synthesis using miScript Reverse Transcription kit (Qiagen, Valencia, CA). Then, using miScript SYBR Green PCR kit (Qiagen, Valencia, CA), the expression levels of RNU6B, miR-32, miR-125a, miR-181c and miR-200c were detected and analyzed in the StepOnePlus Real-Time PCR System (Applied Biosystems, Carlsbad, CA) using specific miRNA primers.

### Analysis of miRNA-regulated genes, pathways, and immunological functions

Potential target gene interactions controlled by dysregulated miRNAs in PTSD patients were analyzed using Ingenuity Pathway Analysis (IPA) tool version 9.0, (Ingenuity Systems, www.ingenuity.com). The dysrgulated miRNAs were uploaded to IPA for analysis. The relationship between dysregulated miRNAs and associated genes of PTSD patients were next analyzed using Cytoscape V.3.0.1 software (Cytoscape Consortium: public databases). Moreover, using ClueGo analysis tool of Cytoscape V.3.0.1 software, relationship between dysregulated miRNAs and associated genes were further analyzed for their role in immunological and/or biological pathways.

### IFN-γ down-regulation by miR-125a

miR-125a precursor (pre-miR125a) and scrambled pre-miRNA (scrambled premiR) at concentrations of 0, 50, or 500 nM (Applied Biosystems, Carlsbad, CA) were introduced into 1×10^6^ PBMC in 100 µl OPTI-MEMI medium (Invitrogen, Carlsbad, CA) by electroporation using BTX ECM830 Elecrto Square Porator (Harvard Apparatus, Holliston, MA) under LV mode at 300v for 10 ms. After culture for two days in 2 mL of X-VIVO 15 medium (Lonza, Walkersville, MD) in the presence of 50 CU/mL IL-2 per well in 24-well plates, IFN-γ release in the culture supernatants from PBMC was determined by enzyme-linked immunosorbent assay (ELISA)using ELISA MAX Set Standard human IFN-γ kit (Biolegend, San Diego, CA) according to the instruction manual.

### Statistical analysis

PTSD patients and normal controls were randomly selected for plasma cytokine measurements, cell culture experiments and miRNA analysis. Experiments for determining percentage of cell populations, plasma cytokines, and Th cell subsets were performed in triplicates. The *p* values for multiple comparisons were calculated based the t-test adjusted with Benjamini & Hochberg (BH) method [46]. The Kruskal Wallis test and Pearson correlation test were used to determine the association between the clinical confounders listed in Table 1 and cell populations, cytokines and miRNAs. Additionally, box plot, scatter plot and bar chart were used to display their relationship. For example, the association between the PBMC counts and clinical scores were investigated using the Kruskal Wallis rank sum test (Box plot in Fig. 1A) or Pearson correlation test (Scatter plot in Fig. 1B). The mean difference between controls and patients illustrated by the bar chart were compared by the t-test, such as shown in Fig. 2A. Furthermore, we illustrated the differences in cytokines or miRNAs of interest by using the heatmap and principle components analysis (PCA) figures. The heatmap was used to describe the correlation between interested variables, such as shown in Fig. 3E, which presented the correlation between PTSD score and cytokines of interested. The PCA was applied to illustrate the difference between the patients and controls. The first three PCA components can account for 60.1% variability of data set [47]. In all analysis, a *p* value of <0.05 was considered to be statistically significant. All statistical analysis was done in R (2.13.2) [47].
